# Autoimmune gastritis masquerading as subepithelial squamous infiltration: a case report

**DOI:** 10.1055/a-2808-7398

**Published:** 2026-02-27

**Authors:** Han Wang, Ziyuan Yu, Zhenyu Chen, Xudan Yang, Xiaogang Liu, Xiao Hu

**Affiliations:** 1 89669 Department of Gastroenterology and Hepatology, Sichuan Provincial Peopleʼs Hospital, School of Medicine, University of Electronic Science and Technology of China, Chengdu, China; 2 89669 School of Medicine, University of Electronic Science and Technology of China, Chengdu, China; 3 198153 Department of Gastroenterology, Nanfang Hospital, Southern Medical University, Guangzhou, China; 4 89669 Department of Pathology, Sichuan Provincial Peopleʼs Hospital, School of Medicine, University of Electronic Science and Technology of China, Chengdu, China

Autoimmune gastritis (AIG) is a chronic immune-mediated disorder with parietal cell destruction, causing hypochlorhydria, hypergastrinemia and the risk of gastric neoplasia 1 2 . Multiple endoscopic signs (corpus-predominant atrophy, sticky mucus and the cast-off skin appearance [CSA]) have been described; yet, a specific endoscopic marker remains unestablished 3 4 .

**Fig. 1:**
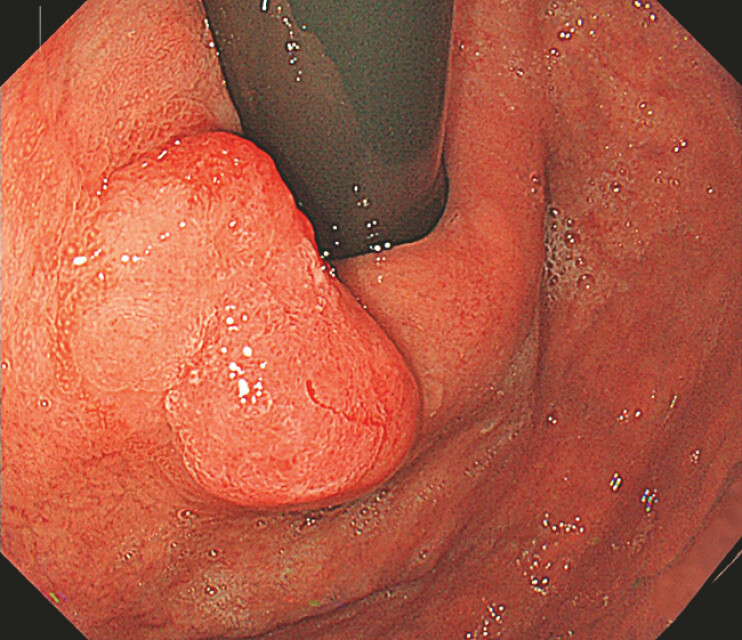
A type 0-IIa lesion at the cardia, measuring approximately 1.5 cm × 3.0 cm, which pathologically confirmed as well-differentiated tubular/papillary adenocarcinoma.

**Fig. 2:**
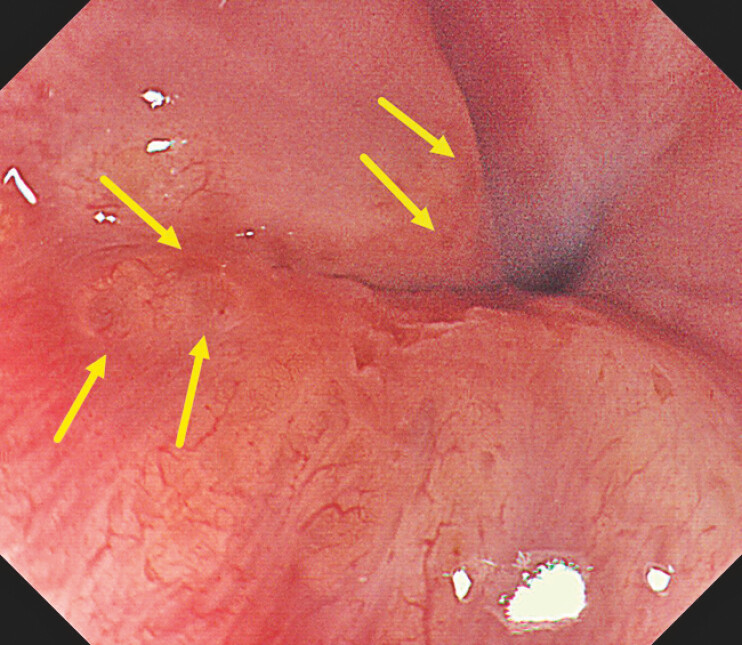
Yellowish granular elevations at the gastroesophageal junction (GEJ) and the translucent “bubble-like” appearance on the white light image (WLI) (indicated by yellow arrows).

**Fig. 3:**
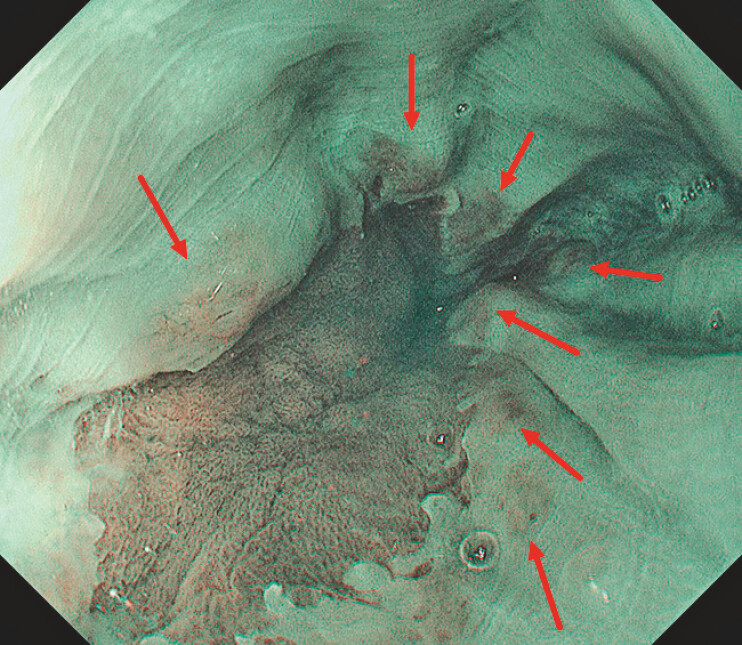
Yellowish granular elevations appear brownish on NBI (indicated by red arrows).

**Fig. 4:**
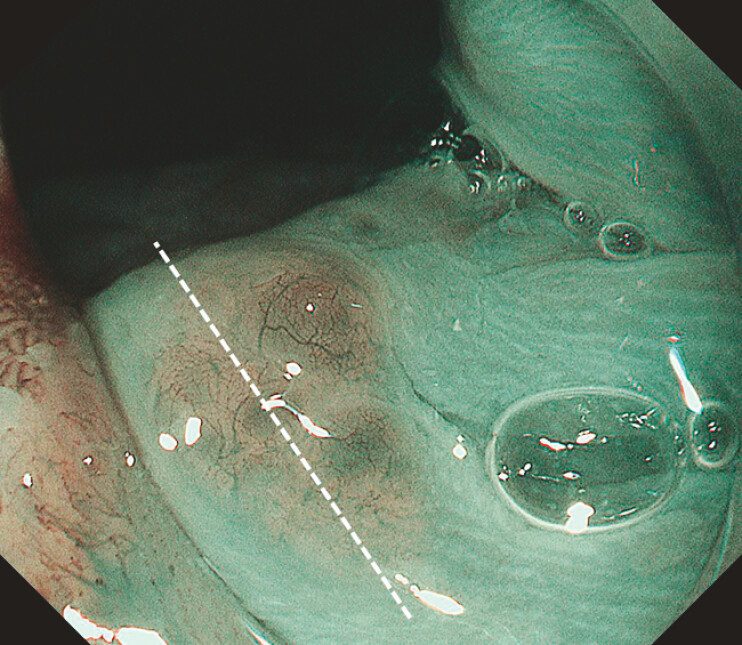
ME-NBI shows the fine reticular vascular structure and the translucent “bubble-like” appearance.

**Fig. 5:**
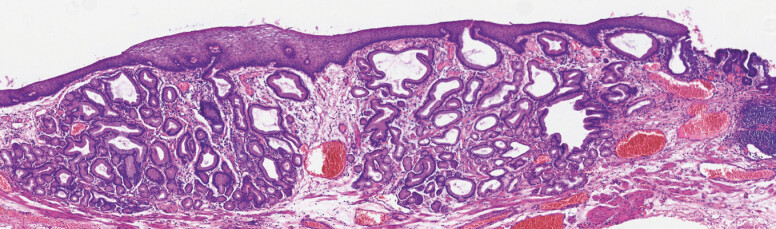
Slide of the white dashed line in Fig. 4 , HE × 20, showing focal thinning of the squamous epithelium and glandular structures in the lamina propria of the esophageal mucosa.

We present the case of a 70-year-old man with prior *Helicobacter pylori* ( *H. pylori* ) eradication. The gastric mucosa exhibits corpus-predominant atrophic gastritis, with a visible vascular pattern, flattened folds in the body and fundus and CSA in the body. Serology confirmed autoimmune gastritis (anti-parietal cell antibody (APCA): 82.97 U/mL, anti-intrinsic factor antibody (AIFA): 6.9 IU/mL, gastrin: 189 pg/mL, pepsinogen I/II: 0.4, and vitamin B12 <83 pg/mL). A type 0-IIa lesion (suspected dysplastic lesion) was observed on the greater curvature of the gastric cardia ( Fig. 1 ). Above the squamocolumnar junction (Z-line), circumferential yellowish granular elevations were observed, with some translucent “bubble-like” appearances on the white light image (WLI; Fig. 2 ), thinning of the squamous epithelium and brownish color on NBI ( Fig. 3 ), and fine reticular microvessels on ME-NBI ( Fig. 4 ). These were initially interpreted as subepithelial squamous infiltration of tumor. Endoscopic submucosal dissection en bloc resection yielded a 3.2 cm × 1.5 cm well-differentiated tubular/papillary adenocarcinoma (tub2>tub1> pap, intestinal phenotype, pT1b). However, the pathology of granulations above the Z-line showed markedly hyperplastic and dilated esophageal cardia glands in lamina propria ( Fig. 5 ), expressing MUC6 and MUC5AC ( Video 1 ).

Autoimmune gastritis masquerading as subepithelial squamous infiltration.Video 1

To validate this finding, we retrospectively analyzed endoscopic records from 20 AIG cases (sequential AIG cases at our institution from May 1 to Nov 30, 2025, with positive APCA/AIFA and histological confirmation) and 20 non-AIG cases (randomly selected from age- and sex-matched patients undergoing gastroscopy in the same period with negative APCA/AIFA). The manifestation was observed in all 20 AIG cases (100%) but absent in controls ( *P* <0.001, Fisherʼs exact test; Video 1 ). This unreported manifestation may be represented as a suggestive endoscopic marker of AIG. While this finding was strongly associated with AIG in our study cohort, we acknowledge that the cardia gland hyperplasia might represent a consequence of hypochlorhydria rather than AIG itself, and further prospective studies are needed to validate its specificity.

Endoscopy_UCTN_Code_TTT_1AO_2AG_3AD
